# Ready or Not…

**DOI:** 10.4269/ajtmh.18-0090

**Published:** 2018-08

**Authors:** B. Jason Brotherton

**Affiliations:** Internal Medicine-Pediatrics, Presbyterian Church of East Africa Chogoria Hospital, Chogoria, Kenya

Volume and variety were the norm inside of the historic walls of the Los Angeles County Hospital, providing a robust clinical foundation to propel the avid learner out into the world of medical practice. Training at Mother County, as she was affectionately referred to, made me ready to handle a multitude of things—I thought I was ready for anything.

It only took a single patient in my first week at our hospital in rural Kenya to shine a bright spotlight on how unprepared I really was for practicing medicine in this new setting.

When the intern called late one night about a 10-day-old baby girl in septic shock, I was ready to put my training into action. Having spent a considerable amount of time caring for critically ill patients, treating sepsis and septic shock was second nature. Therefore, when the anti-inflammatory and pro-inflammatory mechanisms raged through her tiny body to wreak havoc on her newborn organs, I knew that quick antibiotics and meticulous attention to fluid balance would be of utmost importance.

Everything started to come together beautifully in my mind: Careful hydration, frequent auscultation of the lungs, palpation for a descending liver that would indicate over hydration, and continually counting the respiratory rate; evaluation of electrolytes and renal function to assess for evidence of damage; adjustments to her fluids; and the use of intravenous calcium, glucose, and insulin, or even luminal binders to play a proverbial game of musical cations would keep her little body running. I would use the calcium to stabilize her tiny heart cells, while using glucose, followed by insulin to maneuver the potassium back into the cells, hoping her lungs and kidneys would function in concert to rid her body of the accumulating acid. In my mind, I knew exactly what I needed to do to try to help this little baby. The patients, nurses, and attending physicians of Mother County had taught me well.

When I returned to reality equipped with my game plan, I quickly realized that my mental calisthenics were all theory, and I was far from ready.

I had no idea how to monitor and adjust her electrolytes when I could only check the baby’s laboratories, at best, once per day. I had no frame of reference for having nothing more than glucose and insulin to manage her initial hyperkalemia.

Dealing with her worsening respiratory distress was another beast all together. The piecemeal face mask that I held in place seemed to have more moving parts than a pinball machine. Holding the breathing apparatus together took both hands, leaving me defenseless to ward off a kamikaze bird that found its way into the treatment room of our pediatric ward and was feverishly trying to exit. I stood there, desperately trying to help the baby breathe, while avoiding the dive-bombing bird and waiting for the intern to traverse the hospital property to find our lone continuous positive airway pressure machine and mask.

The lost battle to the reckless bird was only an omen of things to come.

When the sun came up and my tiny patient’s condition was not improving, I was reluctantly ready to transfer her to the Big City Hospital (BCH) for a higher level of care. I had never been in a position of needing to send a patient to another facility. I had always worked for the hospital that provided the higher level of care. Now the shoe was on the other foot. Knowing we did not have much time, I quickly got over my pride and was ready to send her to BCH.

However, there was no separate ambulance and team to take her, as there would have been in the United States. I was not ready to ride in the back of a hulled-out old Land Cruiser, holding the baby in my arms for the entire 4-hour journey. Because there was no stretcher, isolette, or seat belts, holding the baby myself seemed like the best option. I wished I could have shared this duty with the kind nurse who volunteered to go with me, but the windy mountain road and her proclivity for car sickness resulted in 4 hours of violent vomiting.

Pulling into the parking lot of BCH with a live baby, a non-puking nurse, our flummoxed driver, and myself all intact with age-appropriate pulses was a victory, albeit short-lived.

Before our arrival, I was under the impression that we were “transferring” our baby to BCH. At least that is how it sounded over the phone. I thought our young baby would be admitted and seen by a nephrologist immediately because despite our best efforts, her creatinine and potassium were both 10, indicating severe renal failure and the potential for cardiac arrest. I was not ready to be told that we were given the wrong information and needed to go to their emergency department for triage like everyone else.

After getting her to the emergency department, I argued for nearly 2 hours with the admitting clinician about what needed to happen next. I adamantly exclaimed she had oliguric renal failure with refractory acidosis and hyperkalemia, clearly indicating the need for dialysis. In my tired and melting mind, that seemed pretty straightforward.

After leaving the hospital frustrated and uncertain if I had been heard, I replayed the scenario hundreds of times in my head. Did I do the right thing? Was my management right, or even good enough? Why would they not listen to me? Should they have listened? I had no real idea how to effectively communicate in this cultural context; the ink was still wet on my visa.

My fervor for advocacy and sense of urgency for needing to do something more for this baby caused me to overlook an important aspect of communication in this culture. In a shame-based culture, confrontation and vehement public disagreement will fall on deaf ears and get you nowhere. Big City Hospital was the referral center for this entire region of Africa. They had to decide the appropriate use of their more substantial, but still limited, resources. I wanted them to use the resources that they had, and we did not, to help this baby.

Still, I desperately wanted to know how it would work out.

Two weeks later while buying produce in the market, I felt a tap on my shoulder. It was the baby’s mother.

“Daktari, do you remember me?” she asked.

When I saw her, I was completely ready to hear how her baby had received dialysis and was improving. I was far from ready to hear how she was in BCH for 3 days and never once received dialysis. When finally taken to the operating room on the fourth day of admission for insertion of the dialysis catheter, she arrested and died on the operating table.

While I know the market continued buzzing, for me the bustling, rural market suddenly became quiet enough to hear a single pin drop. In that moment, I felt like we were immediately placed in a vacuum. I was disappointed and felt like a failure.

I was ready for her to be angry, hurt, and express her disappointment in our ability to care for her young daughter. I waited in anticipation for the deluge of all the things I likely would have said if our roles were reversed.

But that is not what happened. My training had prepared me for her to be angry, for her to expect that her daughter should be alive and should have received only the best care. My training had not prepared me for her to say thank you and then hug me.

Amid her pain and loss and suffering, she offered me something that I so often am ready to receive, but not ready to extend: grace and mercy. In that moment, I was not her daughter’s doctor, but a fellow human who cared for her child and was hurting with her in hearing the tragic news.

That was over 2 years ago, and there is not a day that goes by that I do not reflect on those events. I recall my inexperience and naivete at the time. I perseverate on what we did and what we could have done differently. Even though I had seen sepsis and septic shock plenty of times in training, I had not experienced it here. I had read countless times about how sepsis and septic shock are major killers in resource-limited settings. However, epidemiologic data became real that day. Having gained more experience working in this setting, I realize that dialysis from the minute we stepped into BCH may not have saved her young life.

Putting that aside, what has been tattooed on my soul is that learning to provide excellent patient care in the resource-limited context requires insights far beyond those provided by training in any program or the pages of any book. It also takes humility, flexibility with your limitations, a willingness to learn cultural intricacies, and effective cross-cultural communication. These lessons can only be gleaned after being fully open and engaged with the community around you.

